# Machine Learning Algorithms Highlight tRNA Information Content and Chargaff’s Second Parity Rule Score as Important Features in Discriminating Probiotics from Non-Probiotics

**DOI:** 10.3390/biology11071024

**Published:** 2022-07-07

**Authors:** Carlo M. Bergamini, Nicoletta Bianchi, Valerio Giaccone, Paolo Catellani, Leonardo Alberghini, Alessandra Stella, Stefano Biffani, Sachithra Kalhari Yaddehige, Tania Bobbo, Cristian Taccioli

**Affiliations:** 1 Department of Neuroscience and Rehabilitation, University of Ferrara, Via L. Borsari 46, 44121 Ferrara, Italy; bgc@unife.it; 2 Department of Translational Medicine, University of Ferrara, Via L. Borsari 46, 44121 Ferrara, Italy; nicoletta.bianchi@unife.it; 3 Department of Animal Medicine, Production and Health (MAPS), University of Padua, Via F. Marzolo 5, 35131 Padua, Italy; valerio.giaccone@unipd.it (V.G.); paolo.catellani@unipd.it (P.C.); leonardo.alberghini@unipd.it (L.A.); sachithrakalhari.yaddehige@studenti.unipd.it (S.K.Y.); cristian.taccioli@unipd.it (C.T.); 4 Consiglio Nazionale delle Ricerche (CNR), Istituto di Biologia e Biotecnologia Agraria (IBBA), Via Edoardo Bassini 15, 20133 Milano, Italy; alessandra.stella@ibba.cnr.it (A.S.); stefano.biffani@ibba.cnr.it (S.B.); 5 Department of Agricultural and Environmental Sciences, University of Milan, Via Celoria 2, 20133 Milan, Italy

**Keywords:** probiotics, Machine Learning, tRNA, Chargaff’s Second Parity rule, Shannon’s Entropy

## Abstract

**Simple Summary:**

Probiotics are a group of beneficial microorganisms that are symbionts of the human gut microbiome. The identification of new probiotics is therefore of paramount importance from both public health and commercial perspectives. In this study, we show for the first time that Artificial Intelligence algorithms can identify novel probiotics and also discriminate them from pathogenic organisms in the human gut. We were also able to determine the information content within tRNA sequences as the key genomic features capable of characterizing probiotics.

**Abstract:**

Probiotic bacteria are microorganisms with beneficial effects on human health and are currently used in numerous food supplements. However, no selection process is able to effectively distinguish probiotics from non-probiotic organisms on the basis of their genomic characteristics. In the current study, four Machine Learning algorithms were employed to accurately identify probiotic bacteria based on their DNA characteristics. Although the prediction accuracies of all algorithms were excellent, the Neural Network returned the highest scores in all the evaluation metrics, managing to discriminate probiotics from non-probiotics with an accuracy greater than 90%. Interestingly, our analysis also highlighted the information content of the tRNA sequences as the most important feature in distinguishing the two groups of organisms probably because tRNAs have regulatory functions and might have allowed probiotics to evolve faster in the human gut environment. Through the methodology presented here, it was also possible to identify seven promising new probiotics that have a higher information content in their tRNA sequences compared to non-probiotics. In conclusion, we prove for the first time that Machine Learning methods can discriminate human probiotic from non-probiotic organisms underlining information within tRNA sequences as the most important genomic feature in distinguishing them.

## 1. Introduction

The term “probiotics” refers to live microorganisms that have been shown to exert beneficial functions on human beings when ingested. In particular, if probiotics (usually bacteria but also eukaryotic organisms such as yeasts) are contained in food in a sufficiently large amount, they are able to reach the human gut and promote a balancing action on the intestinal microflora. Indeed, the concept of probiotics, which is a term derived from the ancient Greek “pro-bios”, i.e., pro-life, was coined in 1908, when the Nobel Prize winner Elie Metchnikoff hypothesized that the longevity of Bulgarian farmers was linked to high consumption of fermented milk [1]. Since that time numerous bacteria have been classified as probiotics. In order to consider a microorganism as beneficial for health, its phenotypic characteristics must be confirmed by scientific evidence obtained from high-quality clinical studies conducted on an adequate number of subjects under controlled and randomized sampling. Unfortunately, studies of this type are relatively recent and limited in number; nevertheless, they have led to the characterization of dozens of bacterial species considered beneficial and therefore marketed as probiotics.

Probiotics are of paramount importance not only for individuals with intestinal diseases but also for those with immune deficiency or autoimmune disorders. In fact, with regard to gastrointestinal problems such as constipation and diarrhea, the efficacy of *Lactobacillus casei rhamnosus Lcr35*, which increases the weekly number of evacuations, has long been demonstrated [2]. *Lactobacillus casei Shirota*, on the other hand, is very effective in reducing severe constipation [3], whereas *Lactobacillus rhamnosus GG* (LGG) has been shown to be beneficial improving peristalsis and decreasing diarrhea associated with antibiotics intake, especially in children when they are infected with Rotavirus and *Clostridium difficile* [4]. Probiotics are also used in irritable bowel syndrome, which strongly affects the quality of life in a wide fraction (3 to 25%) of the world population [5]. For this disorder, characterized by recurrent abdominal pain and altered bowel function often associated with bloating and flatulence, there is no real effective treatment. In this case, *Bifidobacterium infantis* and *L. rhamnosus LGG* appear to play an effective role in alleviating the symptoms [6,7,8]. Recently, a potential beneficial effect has been highlighted in the case of ulcerative colitis in patients receiving the bacteria strain *Escherichia coli Nissle* (EcN). Indeed, the combined administration of probiotics containing this microorganism together with the drug Mesalazine seems to increase the probability of success of the treatment [9,10]. Preliminary studies have also shown that the use of probiotics increases the likelihood of suppressing *Helicobacter pylori* infection, which is a health problem in both industrialized and developing countries [11]. Probiotics are not only effective for human intestinal health but also for the immune system. In fact, research in the field of molecular biology and clinical medicine has highlighted the effects of probiotics on lymphocytes and immunoglobulin production [12]. For example, flu and cold symptoms seem to decrease when substantial use of probiotics is implemented [13,14]. Furthermore, probiotics play a role in the prevention of allergic diseases, such as rhinitis [15], and infantile atopic eczema [16].

Given the outstanding importance of probiotics for human health and the absence of fast while accurate selection processes able to discriminate probiotics from non-probiotics, in the present study we developed a Machine Learning (ML) workflow to characterize probiotic bacteria on the basis of their genomic features. ML are Artificial Intelligence algorithms that have the potential to exploit datasets of different size to predict future information based on learning from Past Data, and to discriminate subsets of information. ML algorithms are currently used in numerous fields of science ranging from medicine, pharmacology, finance and arts. In the food sciences, the genomes of bacteria living or infecting gut have been used in several emerging applications to automatically learn very important information such as antibiotic resistance prediction, detection of foodborne outbreaks, possible source of pathogens and risk assessment [17]. In this study, we provide a new method able to detect new probiotic microorganisms, through four ML algorithms, obtaining the most important genomic features that discriminate human probiotic bacteria from non-probiotics. One of the main difficulties to the study of human intestinal probiotics is the small number of these organisms identified today and the absence of accurate and predictive models, and we believe that the development of bioinformatic methods and especially the use of ML techniques can greatly improve research in this field. Indeed, many of the laboratory systems used today are limited by technical complexity and expensiveness, which also limit their practical applications. In this respect, ML and Artificial Intelligence became important computational tools for discovering trends and synergies in large datasets for validation by conventional analytical techniques. Our method involves the use of highly adaptable, trainable algorithms designed to take into account genomic information easily obtained from public databases. Such computational results can help researchers assess the characteristics common to probiotic and to non-probiotic organisms being able to identify new bacterial species possibly used in the biomedical and pharmaceutical fields. In fact, researchers can experimentally test the predictions and validate them with respect to the fundamental characteristics able to discriminate symbiont from pathogenic organisms in the human gastrointestinal tract. To date, ML tools have not yet been used to identify new probiotics, and therefore this work is novel and of considerable interest at the basic and industrial research level.

## 2. Materials and Methods

### 2.1. Dataset

In our dataset we included only bacteria with a completely sequenced genome and good quality annotation, i.e., including complete genomic information for CDS (Coding DNA Sequences), rRNA, tRNA, and mRNA genomic elements. Only bacteria that live in or infect the human gut were considered. In particular, we have excluded bacteria living in the oral, respiratory, urinary, or reproductive tracts, such as *Staphylococcus aureus*, *Staphylococcus salivarius*, *Weissella cibaria*, *Eikenella corrodens*, *Enterococcus avium*, and *Privotella denticola*. In addition, excluded from the analysis were bacteria that colonize non-human intestine (e.g., *Lactobacillus kunkeei* that lives in *Apis mellifera* intestine). The analyzed bacterial genomic data were obtained through GBRAP (GenBank Retrieving, Analyzing and Parsing software) tool [18] using the NCBI ftp bacteria genome database (https://ftp.ncbi.nlm.nih.gov/genomes/refseq/bacteria/ on 31 January 2022). GBRAP was used to download microorganism GenBank files and calculate several genomic scores of both the entire genome and their constitutive elements, such as those encoding for rRNA, tRNA, and genes. The final dataset included 61 genomic features for a total of 89 bacterial organisms, labelled as probiotic or non-probiotic (“outcome” dataset column). The species included in our dataset were validated by scientific experiments published in peer-reviewed journals and manually checked by the authors. Of the 89 records, a subset of 77 already confirmed as probiotic or non-probiotic was used for model training based on the respective relationships between outcome and genomic characteristics; a subset of 12 records was excluded from model building and used as a test set. In particular, the training set included 44 probiotics (7 Bacillales, 9 Bifidobacteriales, 1 Eubacteriales, 26 Lactobacillales, and 1 Propionibacteriales) and 33 non probiotics (2 Acidaminococcales, 5 Bacteroidales, 1 Burkholderiales, 1 Campylobacterales, 12 Clostridiales, 2 Desulfovibrionales, 7 Enterobacterales, 1 Lactobacillales, 1 Pseudomonadales, and 1 Veillonellales). The test set of 12 records, on which we focused in predicting the probiotic/non-probiotic status, included information of 9 bacteria that are currently studied to be marketed as probiotics but whose beneficial characteristics on human health are not yet confirmed (2 Bacteroidales, 1 Eubacteriales, 5 Lactobacillales, and 1 Verrucomicrobiales). Further, three non-probiotic bacteria that are well known to cause diseases of the gastrointestinal human tract (*Rickettia prowazekii*, *Yersinia pseudotuberculosis*, and *Vibrio cholerae*) were added to the test set. Inclusion of the three dangerous bacterial species was done to test the goodness of our model. In fact, the genome size and number of CDS vary in a range of 2.9 Gbp (Giga base pairs) and 2503 genes for non-probiotics, which is included in the range of 3.2 Gbp and 2773 genes of probiotics microorganisms, meaning that the characteristics of these two groups are similar and comparable. Moreover, in the test set we included not only Lactobacillus and Bifidobacteria, which are typically probiotics, but also other taxonomic orders of bacteria such as Verrucomicrobiales and Eubacteriales. Detailed information about the bacterial species included in the dataset can be found in File S1.

### 2.2. Features Encoding

Briefly, among the features in our dataset (see Supplementary Material), the total number of CDS, rRNA, tRNA and non-coding RNA (ncRNA) elements present in the genome were reported. Moreover, we have added the total number of each base and its frequency in each genomic element (CDS, rRNA, tRNA and ncRNA) for the entire sequence of the genome. Topological entropy, Shannon’s Entropy, and Chargaff’s scores (calculated using 2 different approaches, i.e., ct by C. Taccioli or pf by P. Fariselli) were obtained for the genomic sequence, as well as for CDS, rRNA, tRNA and ncRNA sequences. Chargaff’s score [19] describes the ability of a DNA sequence to respect the Chargaff’s Second Parity Rule (CSPR). CSPR states that Adenines are equal in number to Thymines within each genome (excluding animal mitochondria and some single stranded viruses) at the single strand level, as well as Cytosines are equal to Guanines. Any variation from this rule usually denotes an evolutionary force affecting the analyzed DNA sequence that is against the randomicity of a nucleotide molecule. A ct Chargaff’s score of 1 calculated on a DNA sequence means that the CSPR is perfectly complied, or in other words the number of A equals the number of T and the number of C equals the number of G and therefore no evolutionary force has worked on that sequence. pf Chargaff’s score is the same type of measure, but it is not normalized on the length of the sequence and the scale is inverted in the sense that the CSPR is perfectly complied when a value is close to 0. Shannon’s Entropy is the amount of information contained in or provided by an information source, which can be a text written in a given language, an electrical signal or a coding message within a DNA or RNA molecule. A high information content in a DNA sequence has a Shannon’s Entropy of 2 (e.g., ATGC), while a Shannon’s score of 0 (e.g., AAAA) indicates a low information content. Topological entropy, as defined recently [20], is somehow similar to Shannon’s Entropy but more focused on DNA sequences. All formulas and detailed information of all analyzed genomic features are fully explained in File S1.

### 2.3. Recursive Feature Selection and Models Training/Testing

Data processing was carried out following Bobbo et al. [21]. Models to predict the probiotic/non-probiotic status using bacterial genomic features were developed using four ML algorithms: Generalized Linear Model (GLM), Random Forest (RF), Support Vector Machines (SVM), and Neural Network (NN). Before construction of the models, a recursive feature selection with a 10-fold cross-validation (CV) repeated 100 times was performed to automatically select a subset of the most predictive features, in order to identify the most parsimonious model with greatest prediction accuracy. A stratified 10-fold CV repeated 1000 times was then applied to train and validate the models. In particular, the train set was randomly divided into 10 subsets of equal size. Within each of the 10 iterations, nine subsets were used to train the models and one to validate their predictive ability. The entire 10-fold CV was repeated 1000 times, for a total of 10,000 iterations. Data standardization was carried out within CV. Data analysis was performed using Caret v.6.0-86 [22] and Tidyverse v.1.3.1 [23] packages of R software v.4.1.2 [24].

### 2.4. Algorithm Comparison and Evaluation of Predictive Performance on Validation and Test Sets

Accuracy of prediction and Cohen’s Kappa value of each model on validation set were used to compare the four algorithms. The model with the greatest accuracy was then used to assess the features’ importance in determining whether a bacterium is a probiotic or a non-probiotic and to rank the calculated relative importance scores. Outcome prediction on test set was performed using all four ML algorithms and results were analyzed via a confusion matrix, in order to calculate several metrics for comparison (e.g., accuracy, sensitivity, specificity, precision, Cohen’s Kappa value, F1 score, as well as false positive, false negative and total error rates). The pROC package v.1.17.0.1 [25] of R was adopted to calculate the area under the receiver operating characteristic curve (AUC). Finally, as an additional metric to evaluate the classification’s quality, the Matthew’s Correlation Coefficient (MCC) was calculated. Performance evaluation metrics were calculated as follows:

$$\mathrm{Accuracy}=\frac{TP+TN}{TP+TN+FP+FN}$$

(1) $$\mathrm{Cohen}’s\;\mathrm{Kappa}=\frac{2\times \left(TP\times TN-FN\times FP\right)}{\left(TP+FP\right)\times \left(FP+TN\right)+\left(TP+FN\right)\times \left(FN+TN\right)}$$

$$\mathrm{Sensitivity}=\frac{TP}{TP+FN}$$

$$\mathrm{Specificity}=\frac{TN}{TN+FP}$$

$$\mathrm{Precision}=\frac{TP}{TP+FP}$$

$$F1\;\mathrm{score}=\frac{2TP}{2TP+FP+FN}$$

$$\mathrm{MCC}=\frac{TP\times TN-FP\times FN}{\sqrt{\left(TP+FP\right)\times \left(TP+FN\right)\times \left(TN+FP\right)\times \left(TN+FN\right)}}$$

where *TP* is true positive, *TN* is true negative, *FP* is false positive and *FN* is false negative.

## 3. Results

### 3.1. Recursive Feature Selection

**Figure 1:**
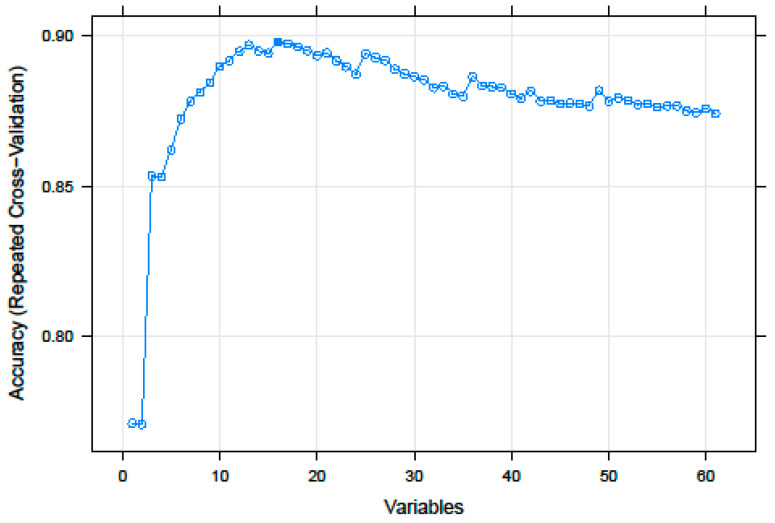
Results of the recursive feature elimination incorporating 1 to all investigated features. An RF analysis was conducted to predict the probiotic/non-probiotic status. The number of features included in the model and the accuracy of prediction are shown on the x-axis and on the y-axis, respectively.

Before model training, a recursive feature selection was applied to reduce their number, removing possible uninformative data. Out of 61 features, only 16 were included in the most parsimonious and performant model, which reached a prediction accuracy of 90.9% (Figure 1). 

The 16 features used for training the model are reported in Table 1. In particular, bp_genome_total, bp_genA and bp_gen_T are genomic features that correspond to the total number of base pairs, the number of Adenine, and number Thymine, the frequency of Guanine and the Shannon’s Entropy, respectively. On the other hand, n_cds_total, bp_cds_total, bp_cdsA, bp_cdsG, bp_cdsT, cds_chargaff_score_ct, cds_chargaff_score_pf, and cds_shannon_score are CDS features corresponding to the total number of CDS elements, the total number of CDS base pairs, the total number of CDS Adenines, the total number of CDS Guanines, the total number of CDS Thymines, the CDS Chargaff’s score (both ct and pf methods) and CDS Shannon’s score, respectively. Another class of features selected by the most parsimonious and performant model are those referred to tRNA elements. The tRNA_chargaff_score (both ct and pf) and tRNA_shannon_score are measures describing the Chargaff’s score within the total sequence of tRNA elements and Shannon’s score calculated on the total sequence of tRNA elements, respectively.

### 3.2. Algorithm Comparison and Evaluation of Predictive Performance on Validation Set

Evaluation and comparison of ML algorithms’ performance in predicting the probiotic/non-probiotic status on the validation set was based on accuracy and Kappa value (Table 2). All four algorithms had an accuracy of prediction above 90%, with NN reaching the greatest value (95.1%), followed by SVM (94.8%). The NN and SVM were characterized also by the greatest Kappa values (0.900 and 0.895, respectively).

**Figure 2:**
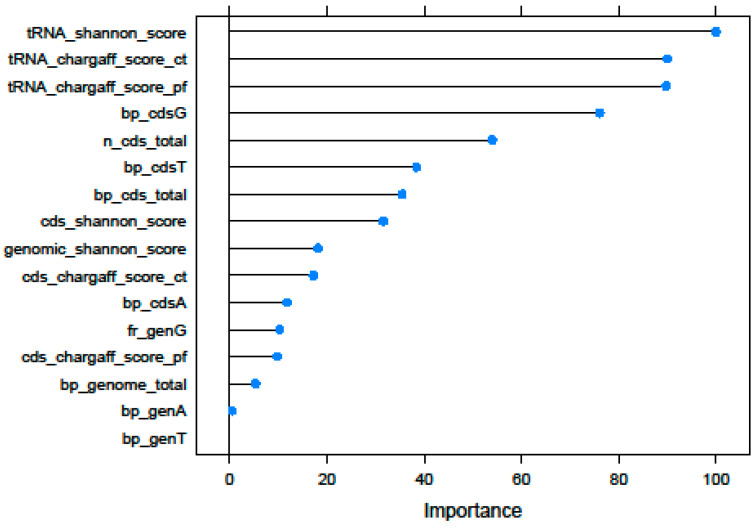
Feature importance plot showing the ranking of the selected features for the prediction of the probiotic/non-probiotic status, using NN as predictive method. Detailed information of the genomic features is fully explained in File S1.

Results of the feature importance analysis, which was performed using NN as a predictive method, revealed that three tRNA-related traits (tRNA_shannon_score, tRNA_chargaff_score_ct, and tRNA_chargaff_score_pf) were the most important features for outcome prediction on the validation set, followed by bp_cdsG and n_cds_total (Figure 2).

### 3.3. Algorithm Comparison and Evaluation of Predictive Performance on Test Set

**Figure 3:**
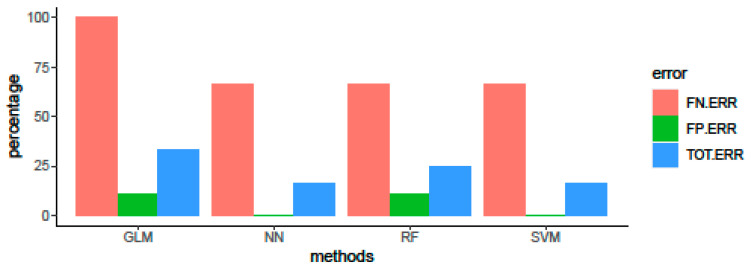
False negative error (FN.ERR), false positive error (FP.ERR) and total error (TOT.ERR) in predicting the probiotic/non-probiotic status on the test set of four ML methods: GLM, RF, SVM and NN.

Metrics for the comparison of ML algorithms predicting performance on the test set are reported in Table 3. The SVM and NN were confirmed as the best methods to predict the probiotic/non-probiotic status, with an accuracy of prediction of 83.3%. In particular, both algorithms correctly classified the three pathogenic bacteria (*Rickettia prowazekii*, *Yersinia pseudotuberculosis*, and *Vibrio cholerae*) as non-probiotics, thus showing no false positive errors (Figure 3) and a specificity of 1 (Table 3). 

Two out of the nine novel possible but not yet confirmed probiotics (*Bacteroides fragilis* and *Bacteroides thetaiotaomicron*, both belonging to the order Bacteroidales) were classified as non-probiotic by all four algorithms. The SVM and NN had also the greatest Kappa value (0.636) and F1 score (0.875), MCC ranged from 0.293 (GLM) to 0.683 (SVM and NN). Finally, SVM and NN showed the greatest ability to distinguish between probiotic and non-probiotic also according to AUC (0.815 for SVM and NN versus 0.704 for RF and 0.630 for GLM).

## 4. Discussion

In this work, we have demonstrated how using ML gives the ability to discriminate between probiotic and non-probiotic bacteria, with possible useful repercussions both in scientific research and in the field of food technology. We considered a dataset comprising 89 records of prokaryotic microorganisms, 77 of which were used for training and validation of the models, whereas the other 12 records were used for testing the predictive ability of our statistical models. Among the four algorithms considered, the NN proved to be the best performing in discriminating these two groups in both validation and testing analysis. The features that most discriminate probiotics from non-probiotics are the Chargaff’s scores (both ct and pf) and Shannon’s Entropy when calculated using tRNA sequences. In particular, probiotics have a lower ct Chargaff’s score (and consequently a higher pf score) than non-probiotics (*p*-value two-tailed test ≤ 0.05; data not shown) and therefore a decreased value of CSPR conformity which means that an evolutionary force has acted considerably on these sequences. This might be due to more pressing environmental conditions of symbiosis with human intestine. This higher content in information of probiotics is also validated by the fact that Shannon’s Entropy is higher in probiotics than in non-probiotics or non-symbionts (*p*-value two-tailed test ≤ 0.05; data not shown). Shannon’s Entropy and Chargaff’s scores are not usually correlated, but in this case both show higher information content in the tRNAs of probiotic comparted to non-probiotic bacteria. Apart from having discovered that it is possible to discriminate probiotics by use of ML, a very interesting finding is that tRNAs appear to be the molecules that have probably most evolved among the genomes of symbiotic intestinal organisms. At the moment, we can only hypothesize what could be the possible roles concerning tRNA molecules, due to the scarce references reported in the literature on these topics. The differential evolution of the genetic code could derive from adaptation phenomena of some microorganisms that lead to the alteration of amino acid specificity. For example, the ancestral link between aminoacyl-tRNA synthetase and tRNAs in the translation process can be very strong [26], and could suggest that an evolution of tRNAs could mirror an evolution of a larger biological machinery. For example, methanogens with a very high Cysteine content in their proteins have a high metabolic demand for this amino acid and a highly expressed tRNA-dependent Cysteine biosynthesis pathway [27]. Instead, in other bacteria, a deletion of a component of the transulfursome may lead many species of microorganisms to be auxotrophic for Cysteine due to their inability to synthesize it [28,29], resulting in a likely loss of information on specific tRNAs [26]. Recently, tRNA molecules with non-canonical structures have also been discovered [30]. These natural tRNA variants are, however, efficiently utilized during translation by the bacterial system and appear to be associated with functional interaction with enzymatic partners that is consistent with highly efficient evolutionary diversification of tRNAs. The ability to colonize the human gut may have also given these bacteria evolutionary advantages that distinguish them from other microorganisms precisely because of specific evolution of tRNA molecules. This would have allowed probiotics to decrease their genome (average genome size non-probiotics ≃ 4.2 Gbp, average genome size probiotics ≃ 2.7 Gbp) by limiting the number of required genes that is another important feature highlighted by our analysis (average number of CDS non-probiotics ≃ 3.7 Mbp, average number of CDS probiotics ≃ 2.5 Mbp). In fact, optimizing genomic resources decreasing the host machinery molecules might have allowed human gut symbiotic organisms to evolve faster and more effectively compared to other competitors. In contrast, non-probiotics have probably had to cope with often unfavorable environments, so evolutionary processes will have rewarded those genomes containing more genes with antibiotic resistance characteristics and so on. In addition, usually the genes encoding for tRNAs are redundant and some of them might be selectively lost in particular taxonomic orders as a likely consequence of negative selection. Furthermore, the different Chargaff’s and Shannon’s scores of the tRNA highlighted in our analysis could take on several aspects, including the principle of purine ejection along the prevailing phylogeny affecting position 34 of the anticodon loop, the wobble position interfering with protein translation, or even tRNA modification depending on Guanosine replacement by Queuosine incorporation or Inosine use. This has probably led to an evolutionary reduction in ambiguous signals or tRNA adaptation phenomena at ribosome sites [31]. Recent findings have shown that tRNAs may interact with other macromolecules that have no translational functions. An example is represented by a bacterial tRNA capable of synthesizing the pentaglycine bridge of the cell wall in addition to having its own function as an amino acid transporter [32]. Similarly, a human tRNA called Asp-GUC has a variant capable of regulating the gene expression of aspartyl-tRNA synthetase [33], highlighting how tRNAs can have multiple functions within eukaryotic cells. Recent evidence also shows that tRNA expression and tRNA modifications in the host can be strongly driven in a microbiome-dependent manner even by varying the secondary structure of these molecules [34]. While there are some non-canonical tRNAs expressed with fine cellular regulation, other tRNA molecules are proved to act as transcriptional regulators involved in nutrient, stress, or immunity responses [35,36]. Another recent function attributed to tRNA genes is that of interfering with DNA replication and transcription events at the level of chromatin structural organization [37]. For example, genes coding for tRNAs (tDNAs) are recognized by specific factors binding the DHU and TѰCG tRNA loop sequences that can modify chromatin condensation and the access to transcriptional factors. Finally, tDNAs are subjected to insertion of transposable elements that can regulate gene expression [31]. This novel study allowed us to state that the use of ML algorithms is effective in discriminating probiotic bacteria from those that live in human gut as non-symbionts. Thus, all these studies indicate that tRNAs are not only vehicles of amino acids for protein biosynthesis but also have regulatory functions within the cell, so it is not surprising that these genomic elements are the most important in discriminating probiotics from non-symbiont organisms in the human gut. Thus, we were able to identify seven new potentially probiotic organisms (Table 4) out of nine (accuracy > 90%). 

In particular, within the test set, NN (along with SVM) correctly identified three non-probiotic bacteria well known to be harmful to the human gut, while it classified as probiotic seven bacteria that are still being studied to be marketed as supplements (all information about these species is included in File S1). However, it is possible that the two bacteria misclassified as non-probiotics (*Bacteroides fragilis* and *Bacteroides thetaiotaomicron*) might not be real probiotics. In fact, they are both able to cause opportunistic infections of various human tissues due to trauma, transforming them from symbiotic bacteria of the human gut, as they usually are, to harmful bacteria. For this reason, they are considered potential probiotics despite their eventual effect on the human body as opportunistic pathogens.

## 5. Conclusions

In this work, we demonstrated the effective use of four ML algorithms (NN, SVM, GLM, and RF) in discriminating probiotics from other non-symbiotic bacteria and in predicting potential new human symbiotic microorganisms that could be used in the food industry in the near future. Furthermore, we were able to identify those genomic features that allow us to distinguish probiotics from other human bacteria living in or infecting the gut. Surprisingly, these characteristics are the information content or evolutionary message found within tRNAs. These RNA molecules are involved in the transfer of a specific amino acid to the nascent polypeptide chain on ribosomes, thus acting as an adaptor between the genetic language carried by the mRNA and the amino acid sequence of the encoded proteins. It is possible, however, that the function of tRNAs is not merely that of cellular transporters of amino acids. New functions are gradually being discovered and understood, and it is possible that some of their characteristics identified in bacteria may be transferred to the study of eukaryotes, whether in the field of food technology (yeasts, e.g., *Saccharomyces cerevisiae*) or human and animal medicine (gut microbiome). Therefore, in the very near future, the improvement of tRNA-sequencing and enrichment techniques will obviously be crucial for a better definition of bacterial RNA biology in relation to transcriptional and translational regulation of the host and may certainly bring new information and knowledge about these molecules once known only as amino acid transporters but that instead seem to play a fundamental role in cellular regulation. In addition, new bacteria genomes, which will be sequenced in the future, will be of considerable interest, not only for food and basic research, but also because they can be included in the training dataset (see File S1) in order to increase the performance of our ML algorithms. Furthermore, the methods presented here can also be used for other organisms, not only prokaryotic but also eukaryotic, to obtain information on the evolution of both pathogenic organisms and those that have developed biological characteristics useful for cohabitation with the host.

## Figures and Tables

**Table 1:** Selected features (n = 16) identified using the most parsimonious and performant model (prediction accuracy = 90.9%).

Id	Selected Features	Description
Genome	bp_genome_total	Genome size
	bp_genA	Total number of Adenines (within the genome)
	bp_genT	Total number of Thymines (within the genome)
	fr_genG	Frequency of Guanines (number of Guanines divided by DNA total length) within the genome
	genomic_shannon_score	Shannon’s Entropy of total genome sequence
CDS	n_cds_total	Total number of CDS elements (Coding DNA Sequences)
	bp_cds_total	Total number of CDS nucleotides
	bp_cdsA	Total number of CDS Adenines
	bp_cdsG	Total number of CDS Cytosines
	bp_cdsT	Total number of CDS Thymines
	cds_chargaff_score_ct	Chargaff’s Second Parity rule score of total CDS sequence (ct method)
	cds_chargaff_score_pf	Chargaff’s Second Parity rule score of total CDS sequence (pf method)
	cds_shannon_score	Shannon Entropy value of total CDS sequence
tRNA	tRNA_chargaff_score_ct	Chargaff’s Second Parity rule score of total tRNA sequence (ct method)
	tRNA_chargaff_score_pf	Chargaff’s Second Parity rule score of total tRNA sequence (pf method)
	tRNA_shannon_score	Shannon’s Entropy value of total tRNA sequence

**Table 2:** Accuracy and Kappa value to compare methods performance on validation set. Prediction models were developed using four different machine learning methods: GLM, RF, SVM and NN.

Method	Accuracy	Kappa Value
GLM	0.936	0.869
RF	0.941	0.880
SVM	0.948	0.895
NN	0.951	0.900

**Table 3:** Metrics (accuracy and 95% Confidence Interval (CI), sensitivity (Se), specificity (Sp), precision, Kappa value, F1 score, MCC and area under the receiver operating characteristic curve (AUC)) to compare methods performance on test set. Prediction models were developed using four different ML methods: GLM, RF, SVM and NN.

Method	Accuracy	95% CI	Se	Sp	Precision	Kappa	F1 Score	MCC	AUC
GLM	0.667	0.349–0.901	0.667	0.667	0.857	0.273	0.750	0.293	0.630
RF	0.750	0.423–0.945	0.778	0.667	0.875	0.400	0.823	0.408	0.704
SVM	0.833	0.516–0.979	0.778	1.000	1.000	0.636	0.875	0.683	0.815
NN	0.833	0.516–0.979	0.778	1.000	1.000	0.636	0.875	0.683	0.815

**Table 4:** Microorganisms included in the test dataset.

Species	Order	NN Classification
*Rickettsia prowazekii*	Rickettsiales	Non-probiotic (referred as a pathogen in literature)
*Yersinia pseudotuberculosis*	Enterobacterales	Non-probiotic (referred as a pathogen in literature)
*Vibrio cholerae*	Vibrionales	Non-probiotic (referred as a pathogen in literature)
*Bacteroides thetaiotaomicron*	Bacteroidales	Non-probiotic (referred as possible probiotics in literature)
*Bacteroides fragilis*	Bacteroidales	Non-probiotic (referred as possible probiotics in literature)
*Paucilactobacillus hokkaidonensis*	Lactobacillales	Probiotic (referred as possible probiotics in literature)
*Akkermansia muciniphila*	Verrucomicrobiales	Probiotic (referred as possible probiotics in literature)
*Levilactobacillus koreensis*	Lactobacillales	Probiotic (referred as possible probiotics in literature)
*Companilactobacillus ginsenosidimutans*	Lactobacillales	Probiotic (referred as possible probiotics in literature)
*Lactobacillus acetotolerans*	Lactobacillales	Probiotic (referred as possible probiotics in literature)
*Limosilactobacillus mucosae*	Lactobacillales	Probiotic (referred as possible probiotics in literature)
*Intestinimonas butyriciproducens*	Eubacteriales	Probiotic (referred as possible probiotics in literature)

## Data Availability

All genetic data have been submitted to https://github.com/tacclab/probiotics (accessed on 1 April 2022).

## Supplementary Materials

The following supporting information can be downloaded at: https://www.mdpi.com/article/10.3390/biology11071024/s1, Excel files containing the dataset and the validation set are freely available at: https://github.com/tacclab/probiotics accessed on 1 April 2022, File S1: Dataset and validation set [38,39,40,41,42,43,44].
