# Supplementary material for: Machine Learning Algorithms Highlight tRNA Information Content and Chargaff’s Second Parity Rule Score as Important Features in Discriminating Probiotics from Non-Probiotics
Source: Biology (Basel). 2022 Jul 7;11(7):1024. doi: 10.3390/biology11071024 (PMC9311688; doi:10.3390/biology11071024)
Supplement: Supplementary file 1 [file biology-11-01024-s001.zip › biology-1747832-supplementary.pdf]

Dataset column legend

- ID: GenBank identification code;
- name: organism's name;
- taxon: organism taxon belonging;
- bp_genome: total number of bases in the genomic sequence;
- bp_genA: total number of Adenines in the genomic sequence;
- bp_genT: total number of Thymines in the genomic sequence;
- bp_genC: total number of Cytosines in the genomic sequence;
- bp_genG: total number of Guanines in the genomic sequence;
- fr_genA: frequency of Adenine nucleotides in the genomic sequence;
- fr_genT: frequency of Thymine nucleotides in the genomic sequence;
- fr_genC: frequency of Cytosine nucleotides in the genomic sequence;
- fr_genG: frequency of Guanine nucleotides in the genomic sequence;
- genomic_topological_entropy_score: Topological score calculated on genomic sequences using Koslicki's method (Koslicki, D., Bioinformatics, 2011);
- genomic_chargaff_score_pf: Chargaff's second parity rule score calculated on genomic sequences as shown above;
- genomic_chargaff_score_ct: Chargaff's score calculated on genomic sequences using CT method as shown above;
- genomic_shannon_score: Shannon's score calculated on genomic sequences using Shannon's method (Shannon, CE., The Bell Technical Journal, 1948);
- bp_cds_total: total number of bases in the coding sequences;
- bp_cdsA: total number of Adenines in the coding sequences;
- bp_cdsT: total number of Thymines in the coding sequences;
- bp_cdsC: total number of Cytosines in the coding sequences;
- bp_cdsG: total number of Guanines in the coding sequences;
- fr_cdsA: frequency of Adenine nucleobase in the coding sequences;
- fr_cdsT: frequency of Thymine nucleobase in the coding sequences;
- fr_cdsC: frequency of Cytosine nucleobase in the coding sequences;
- fr_cdsG: frequency of Guanine nucleobase in the coding sequences;
- n_cds_plus: number of coding sequences on the + strand of the genomic sequence;
- n_cds_minus: number of coding sequences on the - strand of the genomic sequence;
- n_cds_total: total number of coding sequences in the genomic sequence;

- cds_chargaff_score_pf: Chargaff's second parity rule score calculated on cds sequences as shown above;
- cds_chargaff_score_ct: Chargaff’s score calculated on cds sequences using CT method as shown above;
- cds_shannon_score: Shannon’s score calculated on cds sequences using Shannon’s method (Shannon, CE., The Bell Technical Journal, 1948);
- bp_rRNA_total: total number of bases in the rRNA sequences;
- bp_rRNA_A: total number of Adenines in the rRNA sequences;
- bp_rRNA_T: total number of Thymines in the rRNA sequences;
- bp_rRNA_C: total number of Cytosines in the rRNA sequences;
- bp_rRNA_G: total number of Guanines in the rRNA sequences;
- fr_rRNA_A: frequency of Adenine nucleobase in the rRNA sequences;
- fr_rRNA_T: frequency of Thymine nucleobase in the rRNA sequences;
- fr_rRNA_C: frequency of Cytosine nucleobase in the rRNA sequences;
- fr_rRNA_G: frequency of Guanine nucleobase in the rRNA sequences;
- n_rRNA_total: total number of rRNAs in the genomic sequence;
- rRNA_chargaff_score_pf: Chargaff's second parity rule score calculated on rRNA sequences as shown below;
- rRNA_chargaff_score_ct: Chargaff’s score calculated on rRNA sequences using CT method as shown below;
- rRNA_shannon_score: Shannon’s score calculated on rRNA sequences using Shannon’s method (Shannon, CE., The Bell Technical Journal, 1948);
- bp_tRNA_total: total number of bases in the tRNA sequences;
- bp_tRNA_A: total number of Adenines in the tRNA sequences;
- bp_tRNA_T: total number of Thymines in the tRNA sequences;
- bp_tRNA_C: total number of Cytosines in the tRNA sequences;
- bp_tRNA_G: total number of Guanines in the tRNA sequences;
- fr_tRNA_A: frequency of Adenine nucleobase in the tRNA sequences;
- fr_tRNA_T: frequency of Thymine nucleobase in the tRNA sequences;
- fr_tRNA_C: frequency of Cytosine nucleobase in the tRNA sequences;
- fr_tRNA_G: frequency of Guanine nucleobase in the tRNA sequences;
- n_tRNA_total: total number of tRNAs in the genomic sequence;
- tRNA_chargaff_score_pf: Chargaff's second parity rule score calculated on tRNA sequences as shown above;

- tRNA_chargaff_score_ct: Chargaff's score calculated on tRNA sequences using CT method as shown above;
- tRNA_shannon_score: Shannon's score calculated on tRNA sequences using Shannon's method (Shannon, CE., The Bell Technical Journal, 1948);
- bp_ncRNA_total: total number of bases in the ncRNA sequences;
- bp_ncRNA_A: total number of Adenines in the ncRNA sequences;
- bp_ncRNA_T: total number of Thymines in the ncRNA sequences;
- bp_ncRNA_C: total number of Cytosines in the ncRNA sequences;
- bp_ncRNA_G: total number of Guanines in the ncRNA sequences;
- fr_ncRNA_A: frequency of Adenine nucleobase in the ncRNA sequences;
- fr_ncRNA_T: frequency of Thymine nucleobase in the ncRNA sequences;
- fr_ncRNA_C: frequency of Cytosine nucleobase in the ncRNA sequences;
- fr_ncRNA_G: frequency of Guanine nucleobase in the ncRNA sequences;
- n_ncRNA_total: total number of ncRNAs in the genomic sequence;
- ncRNA_chargaff_score_pf: Chargaff's second parity rule score calculated on ncRNA sequences as shown above;
- ncRNA_chargaff_score_ct: Chargaff's score calculated on ncRNA sequences using CT method as shown above;
- ncRNA_shannon_score: Shannon's score calculated on ncRNA sequences using Shannon's method (Shannon, CE., The Bell Technical Journal, 1948);

Chargaff's second parity rule pf (Piero Fariselli) score calculation

$((\#A-\#T)/(\#A+\#T)) + ((\#C-\#G)/(\#C+\#G))$

where "#" means "number of" and A = Adenines, T = Thymines, C = Cytosines and G = Guanines

Chargaff's second parity rule ct (Cristian Taccioli) score calculation (code in Python 3.8)

$|((\#A/\#T)+(\#C/\#G))/2|, \forall\ \#A<\#T, \forall\ \#C<\#G^*$

where "#" means "number of" and A = Adenines, T = Thymines, C = Cytosines and G = Guanines

* In this formula, the base with the lowest number of nucleotides within the sequence must always be at the numerator.

########### **Pyhton 3.8 code** ############

```
def count_bases(self,sequence):
''' It counts the number of bases in a sequence'''
A=sequence.count("A")
T=sequence.count("T")
```

```
    C=sequence.count("C")
    G=sequence.count("G")
    a=sequence.count("a")
    t=sequence.count("t")
    c=sequence.count("c")
    g=sequence.count("g")
    nA=a+A
    nT=t+T
    nC=c+C
    nG=g+G
    tot=nA+nT+nC+nG
    return [tot,nA,nT,nC,nG]

def chargaff_CT(s):
    ''' Method for Shannon entropy and Chargaff score by C.T '''
    s = str(s).replace("N","")
    nt = count_bases(s)

    if nt[0] !=0 and nt[1] !=0 and nt[2] !=0 and nt[3] !=0 and nt[4] !=0:
        pA = float(nt[1]/nt[0])
        pT = float(nt[2]/nt[0])
        pC = float(nt[3]/nt[0])
        pG = float(nt[4]/nt[0])


        if nt[1] <= nt[2]:
            rAT = nt[1]/nt[2]
        else:
            rAT = nt[2]/nt[1]
        if nt[3] <= nt[4]:
            rCG = nt[3]/nt[4]
        else:
            rCG = nt[4]/nt[3]
        result_ct = (rAT+rCG)/2
    else:
        try:
```

```
    pA = float(nt[1]/nt[0])
except:
    pA = 'NA'

try:
    pT = float(nt[2]/nt[0])
except:
    pT = 'NA'

try:
    pC = float(nt[3]/nt[0])
except:
    pC = 'NA'

try:
    pG = float(nt[4]/nt[0])
except:
    pG = 'NA'

result_ct = 'NA' # Chargaff score by C.T
```

Shannon's entropy formula

$$E = -\sum_i (p(i) \times \log_2(p(i)))$$

Where p(i) is the frequency of each letter in a message (i.e. a nucleotide in case of a DNA sequence

Dataset splitting approach for the creation of train, test and validation sets

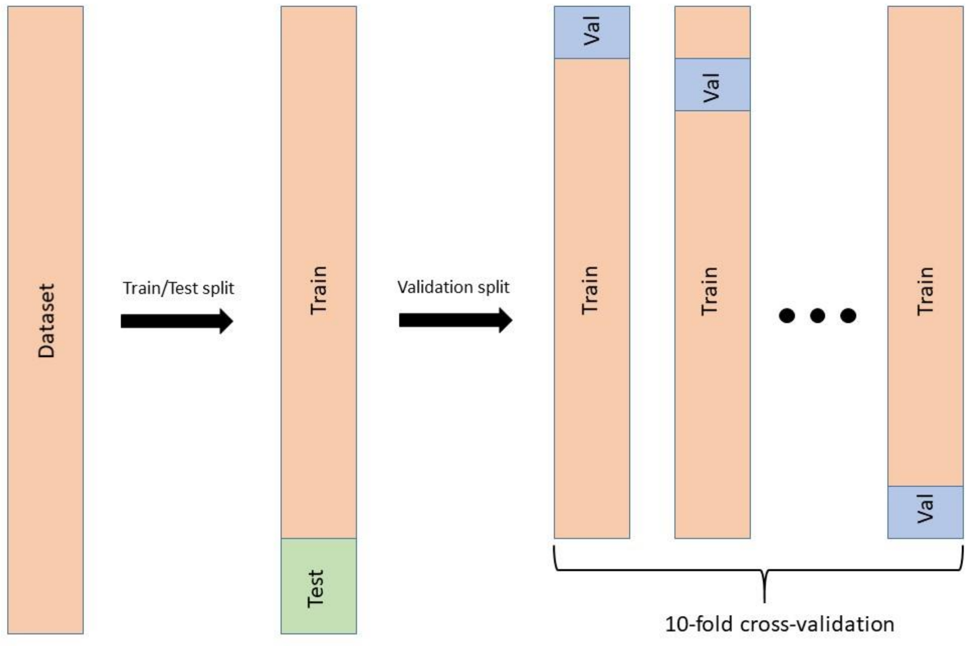

Tuning details of the four machine learning algorithms

Generalized linear models (GLM)

Resampling results across tuning parameters:

alpha	lambda	Accuracy	Kappa
0.10	0.0005571896	0.8849206	0.7540241
0.10	0.0055718962	0.8581349	0.7019226
0.10	0.0557189619	0.7795635	0.5435893
0.55	0.0005571896	0.8992063	0.7872257
0.55	0.0055718962	0.8581349	0.7019226
0.55	0.0557189619	0.7795635	0.5435893
1.00	0.0005571896	0.9357143	0.8689350
1.00	0.0055718962	0.8581349	0.7046894
1.00	0.0557189619	0.7938492	0.5767909

Accuracy was used to select the optimal model using the largest value.
The final values used for the model were alpha = 1 and lambda = 0.0005571896.

Random Forest (RF)

Resampling results across tuning parameters:

mtry	Accuracy	Kappa
2	0.8984127	0.7865635
9	0.9412698	0.8806346
16	0.9412698	0.8806346

Accuracy was used to select the optimal model using the largest value.
The final value used for the model was mtry = 9.
The "rf" method in the Caret package sets the default number of trees at 500.

Support Vector Machines (SVM)

Resampling results across tuning parameters:

C	Accuracy	Kappa
0.25	0.8285714	0.6755822
0.50	0.9482143	0.8945652
1.00	0.9357143	0.8695652

Tuning parameter 'sigma' was held constant at a value of 0.3946823
Accuracy was used to select the optimal model using the largest value.
The final values used for the model were sigma = 0.3946823 and C = 0.5.

Neural Network (NN)

Resampling results across tuning parameters:

size	decay	Accuracy	Kappa
1	0e+00	0.8041667	0.5856711
1	1e-04	0.7662698	0.5073165
1	1e-01	0.7898810	0.5597235

3	0e+00	0.8688492	0.7309351
3	1e-04	0.8291667	0.6332363
3	1e-01	0.8863095	0.7702978
5	0e+00	0.7787698	0.5473316
5	1e-04	0.8765873	0.7487678
5	1e-01	0.9509921	0.8995371

Accuracy was used to select the optimal model using the largest value.
The final values used for the model were size = 5 and decay = 0.1.

Bacteria used in the test set

• ***Lactobacillus mucosae***: it could be a new potential probiotic according to Jiang *et al*. [38];
• ***Intestinimonas butyriciproducens***: it could be a potential probiotic according to Bui et al. [39];
• ***Lactobacillus acetotolerans***: it could be a probiotic according to Tsai *et al*. [40];
• ***Lactobacillus koreensis****: it* could be a potential probiotic according to Sun *et al*. [41];
• ***Bacteroides thetaiotaomicron****: it* could be a potential probiotic according to Chow *et al*. [42];
• ***Akkermansia muciniphila***: it could be a probiotic according to Cani et al. [43];
• ***Bacteroides fragilis***: it might be a potential probiotic according but is also possible [44];
• ***Lactobacillus hokkaidonensis***: Recently new studies [41];
• ***Lactobacillus ginsenosidimutans***: Recently new studies [41];
• ***Rickettsia prowazekii***: it is a well-known bacterium that can become an obligate intracellular parasite, being an aetiological agent of exanthematous typhus and Brill's disease. It multiplies in the gut epithelium of the louse and then releases rickettsiae into the feces. Symptoms of exanthematous typhus are fever, nausea, vomiting, stomach pain and rash;
• ***Vibrio cholerae***: Vibrio cholerae is one of the most clinically relevant and studied bacteria as the aetiological agent of human intestinal cholera. Symptoms of cholera are profuse watery diarrhoea, vomiting, thirst, leg cramps and restlessness or irritability;
• ***Yersinia pseudotuberculosis***: It is a well-known bacterium of the genus Yersinia and is a pathogen. In the evolution from the subacute to the chronic course there are attacks of diarrhoea and non-specific symptoms.
